# The choice of internal fixator for fractures around the femoral trochanter depends on area classification

**DOI:** 10.1186/s40064-016-3206-1

**Published:** 2016-09-08

**Authors:** Hiroaki Kijima, Shin Yamada, Natsuo Konishi, Hitoshi Kubota, Hiroshi Tazawa, Takayuki Tani, Norio Suzuki, Keiji Kamo, Yoshihiko Okudera, Ken Sasaki, Tetsuya Kawano, Naohisa Miyakoshi, Yoichi Shimada

**Affiliations:** 1 Department of Orthopedic Surgery, Akita University Graduate School of Medicine, 1-1-1 Hondo, Akita, 010-8543 Japan; 2 Akita Hip Research Group, Akita, Japan

**Keywords:** Femoral trochanteric fractures, Area classification, Proximal femoral fractures, Internal fixation

## Abstract

**Purpose:**

In femoral trochanteric fractures, fractures whose fracture lines extend to the basal neck or to the subtrochanteric part have high instability. Area classification can identify such instable fractures. The best choices of internal fixators for femoral trochanteric fractures were investigated according to area classification.

**Methods:**

Femoral trochanteric fractures were investigated with respect to area classification. In area classification, the proximal femur is divided into 4 areas with 3 boundary lines: Line-1 is the center of the neck; Line-2 is the border between the neck and the trochanteric zone; and Line-3 links the inferior borders of the greater and lesser trochanters. A fracture in only the third area was classified as type 3; one in the second and third areas was classified as type 2–3.

**Results:**

Of 284 femoral trochanteric fractures, 50.0 % were type 3, 21 % were type 2–3, 22 % were type 3–4, and 7.4 % were type 2–3–4. Cases with cut-out or excessive telescoping of the internal fixator were defined as the Failure-group; 5.3 % of type 3 and 10.9 % of type 2–3 were in the Failure-group only when short femoral nails with a single rag screw were used. On the other hand, there were no Failure-group cases of type 2–3 with double rag screws. Only 1 case involved a long nail for type 3, while a long nail was used in about half of type 3–4 cases (Chi square test: P < 0.0001).

**Conclusions:**

A double rag screw should be considered for type 2–3. A long nail should be considered for type 3–4.

## Background

Femoral trochanteric fracture is a very common injury (Nikkel et al. 2012; Kannus et al. 1996). However, in the so-called femoral trochanteric fracture, a fracture whose fracture line extends to the basal neck or the subtrochanteric part is not rare. A fracture extending from the basal neck to the subtrochanteric part is included among so-called femoral trochanteric fractures. Such fractures have high instability, and the treatment method is different from that for femoral trochanteric fractures within only the trochanteric zone. Nevertheless, we cannot classify such fractures conventionally because the fractures cross the target range of the conventional classification: femoral neck fracture, basicervical fracture, pertrochanteric fracture, or subtrochanteric fracture. Therefore, the choice of internal fixator for such fractures around the femoral trochanter is very difficult. Thus, area classification has been proposed as a comprehensive classification that can identify such fractures (Kijima et al. 2014).

**Fig. 1:**
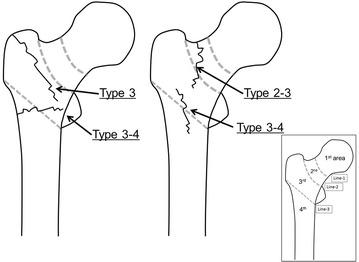
Fractures around the femoral trochanter classified by area classification. Fractures within only the trochanteric part are classified as type 3, fractures extending from the trochanteric part to the basal neck are classified as type 2–3, fractures extending from the trochanteric part to the subtrochanteric part are classified as type 3–4, and fractures extending from the basal neck to the subtrochanteric part are classified as type 2–3–4

In area classification, the proximal femur is divided into 4 areas with 3 boundary lines: Line-1 is the center of the neck; Line-2 is the border between the neck and the trochanteric zone; and Line-3 links the inferior borders of the greater and lesser trochanters. A fracture in only the first area is classified as a type 1 fracture; one in the first and second area is classified as a type 1–2 fracture. In the same way, fractures are classified as type 1, type 2, type 3, type 4, type 1–2, type 2–3, type 3–4, type 1–2–3, type 2–3–4, and type 1–2–3–4 (10 types). In this classification, so-called neck fractures, basicervical fractures, trochanteric fractures, and subtrochanteric fractures are defined by the boundary lines. In addition, area classification can classify the fractures that cross the zones (Kijima et al. 2014). Fractures within only the trochanteric part are classified as type 3, fractures extending from the trochanteric part to the basal neck are classified as type 2–3, fractures extending from the trochanteric part to the subtrochanteric part are classified as type 3–4, and fractures extending from the basal neck to the subtrochanteric part are classified as type 2–3–4 (Fig. 1).

As for almost all so-called femoral trochanteric fractures, osteosynthesis is chosen, and total hip replacement or femoral head replacement is not chosen, unlike for femoral neck fractures (Evans 1951; Reno and Burlington 1958). Most type 3 fractures (fracture line within only the trochanteric part) have recently been treated by a short femoral nail (Bojan et al. 2010; Guo et al. 2015). However, which internal fixator should be chosen for cases in which the fracture line extends from the trochanteric zone to the basal neck or to the subtrochanteric part is unknown.

Therefore, the relationships between the clinical results and the choices of internal fixators for the fractures around the femoral trochanter, area classification type 3 (fracture line within only the trochanteric part), type 2–3 (fracture lines extend from the trochanteric part to the basal neck), type 3–4 (fracture lines extend from the trochanteric part to the subtrochanteric part), and type 2–3–4 (fracture lines extend from the basal neck to the subtrochanteric part), were investigated in this study.

## Methods

The subjects were patients with fractures around the femoral trochanter who were brought to 6 general hospitals from January to December 2014. All patients underwent X-ray and computed tomography (CT) examinations, including 3-dimensional-CT (3DCT).

All procedures performed in this study involving human participants were in accordance with the ethical standards of the institutional committee and with the 1964 Helsinki declaration and its later amendments or comparable ethical standards. Informed consent was obtained from all individual participants included in the study at the last observation which is after July, 2015. Thus, this study is retrospective study.

A total of 482 patients with proximal femoral fractures were seen in the six general hospitals from January to December 2014 (average patient age 81 years (26–108 years); 98 male and 384 female). After having classified these by area classification, only cases classified as type 3, type 2–3, type 3–4, and type 2–3–4 were evaluated. After July, 2015, the orthopedic surgeon of each hospital classified the above patients by area classification, with reference to preoperative X-rays images and CT including 3DCT. There was no need to have several examiners perform classification, because area classification has been shown to have high reliability (Kijima et al. 2014).

The internal fixator used for these cases was investigated. Cases with cut-out or telescoping of the internal fixator of more than 10 mm were defined as the Failure (F)-group. The failure rate was calculated as the percentage of cases in the F-group. The cases without cut-out or telescoping of the internal fixator of more than 10 mm were defined as the N-group.

## Results

**Fig. 2:**
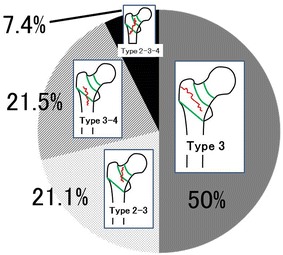
Incidence of fractures around the femoral trochanter classified by area classification. In area classification, 142 cases (50.0 %) are type 3 fractures, 60 cases (21.1 %) are type 2–3 fractures, 61 cases (21.5 %) are type 3–4 fractures, and 21 cases (7.4 %) are type 2–3–4 fractures

**Fig. 3:**
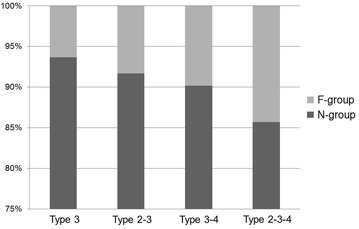
Failure rate of each fracture type. Cases with cut-out or telescoping of the internal fixator of more than 10 mm were defined as the F-group. The failure rate was the percentage of cases in the F-group. Cases without cut-out or telescoping of the internal fixator of more than 10 mm were defined as the N-group. The failure rate is 6.3 % in type 2–3, 8.3 % in type 2–3, 9.8 % in type 3–4, and 14.3 % in type 2–3–4

**Fig. 4:**
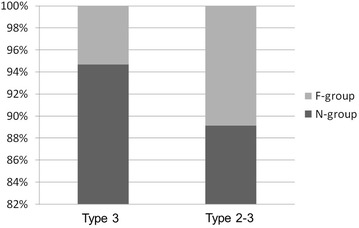
The failure rate of type 3 and type 2–3 fixed with a single rag screw. When type 2–3 fractures are compared with type 3 fractures, the failure rates of type 3 and type 2–3 fractures, which were fixed by an internal fixator with a single rag screw, are 5.3 and 10.9 %, respectively

**Fig. 5:**
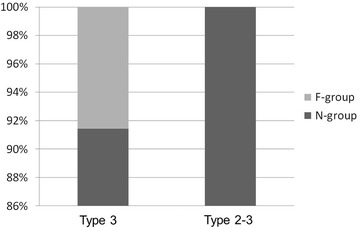
The failure rate of type 3 and type 2–3 fixed with a double rag screw. The failure rates of type 3 and type 2–3 fractures, which were fixed by an internal fixator with a double rag screw, are 8.6 and 0 %, respectively

**Fig. 6:**
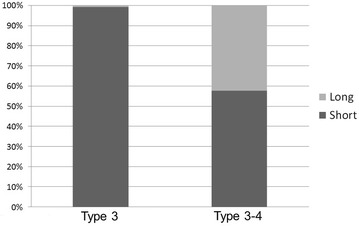
The choice of internal fixator for type 3 and type 3–4. When the choice of internal fixator for type 3–4 fractures is compared with that for type 3 fractures, a long nail was chosen in 42.3 % in type 3–4 fractures, whereas a long nail was chosen in only 1 case (0.8 %) in type 3 fractures; this difference is significant (Chi square test; P < 0.0001)

Overall, femoral trochanteric fractures, namely fractures around area-3 in area classification occurred in 284 of the 482 cases (58.9 %). In area classification, 142 cases (50.0 %) were type 3 fractures, 60 (21.1 %) were type 2–3 fractures, 61 (21.5 %) were type 3–4 fractures, and 21 (7.4 %) were type 2–3–4 fractures (Fig. 2). Average follow-up duration is 4 months (1 month–18 months). Osteosynthesis was performed with the third-generation short Gamma nail or long Gamma nails (Stryker Japan, Tokyo, Japan), Proximal Femoral Nail Antirotation (PFNA) (DePuy Synthes Japan, Tokyo, Japan), and IPT nail system (HOMS giken, Nagano, Japan). The failure rate was 6.3 % in type 3, 8.3 % in type 2–3, 9.8 % in type 3–4, and 14.3 % in type 2–3–4 (Fig. 3). When type 2–3 fractures were compared with type 3 fractures, the failure rates of type 3 and type 2–3 fractures, which were fixed by an internal fixator with a single rag screw, were 5.3 and 10.9 %, respectively (Fig. 4). On the other hand, the failure rates of type 3 and type 2–3 fractures, which were fixed by internal fixators with double rag screws, were 8.6 and 0 %, respectively (Fig. 5). This difference in failure rate between the rate of using single rag screw for type 3 and that for type 2–3 was significant (Chi square test; P = 0.0014). When the choice of internal fixator for type 3–4 fractures was compared with that for type 3 fractures, a long nail was chosen in 42.3 % of type 3–4 fractures, whereas a long nail was chosen in only 1 case (0.8 %) of type 3 fractures; this difference was significant (Chi square test; P < 0.0001) (Fig. 6). However, there was no significant difference in fixation failure between short nail and long nail for type 3–4 (Chi square test; P = 0.0621).

## Discussion

The relationships between clinical results and the choices of internal fixators for fractures around the femoral trochanter, area classification type 3, type 3–4, and type 2–3–4 were investigated in this study. Given the results, double rag screws should be considered for fractures extending from the trochanteric part to the basal neck (type 2–3), and a long nail should be considered for fractures extending from the trochanteric part to the subtrochanteric part (type 3–4).

Femoral trochanteric fractures are very common, and it was apparent that there were fractures whose fracture lines extended to the basal neck or to the subtrochanteric part, without remaining only in the trochanter part. However, there have been no reports that investigated the frequency of such unstable fractures. In this study, it became clear that, among the 284 fractures around the trochanteric part, namely area-3, in 142 cases the fracture line remained only in the trochanteric part, and the remaining half were such unstable fractures whose fracture lines extended to the basal neck or the subtrochanteric part. These are the data provided by classifying femoral trochanteric fractures using area classification. The high reliability of area classification has already been reported (Kijima et al. 2014).

Fractures that included multiple areas had a tendency to include many cases with cut-out of the inner fixator or excessive telescoping. Therefore, it appears that it was necessary to perform osteosynthesis very carefully, especially for the trochanteric part fractures, whose fracture line extended to the basal neck or to the subtrochanteric part. However, the abstract advice to perform osteosynthesis carefully is useless for deciding which internal fixator to use for fractures around the femoral trochanter. In this study, the relationships between the choice of internal fixator and the clinical results were investigated based on area classification. Given the present results, clear advice can now be given: a double rag screw and a long nail should be considered for type 2–3 and type 3–4 fractures, respectively. Therefore, area classification was useful in the choice of internal fixator for fractures around the femoral trochanter.

One of the limitations of this study was that there was no significant difference in fixation failure between short nail and long nail for type 3–4, maybe because the orthopedic surgeons used the long nail for unstable fractures and used short nail for stable fractures. However, if so, the significant difference in the use of long nail favoring type 3–4 over type 3 can be the evidence for the superiority of long nail over short nail for type 3–4. The comparison in fixation failure between short nail and long nail for type 3–4 should be considered in next study which has more large number of fractures.

Another of the limitations of this study were that real instability cannot be evaluated based on area classification. To make a fracture model based on area classification and to evaluate instability of each type is necessary in the future. In addition, a biomechanical experiment to clarify which inner fixator most strongly contributes to improving stability is also needed. The results of the present study need to be confirmed by such studies.

In other words, this study provided important evidence that the comprehensive classification of proximal femoral fractures called area classification is useful for fractures around the femoral trochanter.

## Conclusion

Area classification was useful because it could classify the unstable fractures that cross over the conventional classification range. A double rag screw should be considered for fractures extending from the trochanteric part to the basal neck (type 2–3), while a long nail should be considered for fractures extending from the trochanteric part to the subtrochanteric part (type 3–4). Therefore, area classification was useful also in the choice of internal fixator for fractures around the femoral trochanter.
